# Myelin Basic Protein Attenuates Furin-Mediated Bri2 Cleavage and Postpones Its Membrane Trafficking

**DOI:** 10.3390/ijms25052608

**Published:** 2024-02-23

**Authors:** Evgeniya V. Smirnova, Vladimir I. Timofeev, Tatiana V. Rakitina, Dmitry E. Petrenko, Olga S. Elmeeva, George A. Saratov, Anna A. Kudriaeva, Eduard V. Bocharov, Alexey A. Belogurov

**Affiliations:** 1 Shemyakin and Ovchinnikov Institute of Bioorganic Chemistry, Russian Academy of Sciences, 117997 Moscow, Russia; smirnova.evgeniya@gmail.com (E.V.S.); taniarakitina@yahoo.com (T.V.R.); anna.kudriaeva@gmail.com (A.A.K.); 2 National Research Centre “Kurchatov Institute”, 123182 Moscow, Russia; tostars@mail.ru (V.I.T.); dmitry.e.petrenko@gmail.com (D.E.P.); 3 Department of Chemistry and Technology of Biologically Active Compounds, Medical and Organic Chemistry Named after N.A. Preobrazhensky, Lomonosov Institute of Fine Chemical Technologies, MIREA—Russian Technological University, 119571 Moscow, Russia; elmeevaolia@gmail.com; 4 Phystech School of Biological and Medical Physics, Moscow Institute of Physics and Technology (National Research University), 141701 Dolgoprudny, Moscow Region, Russia; 5 Department of Biological Chemistry, Federal State Budgetary Educational Institution of Higher Education “Russian University of Medicine” of the Ministry of Health of the Russian Federation, 127473 Moscow, Russia

**Keywords:** myelin basic protein (MBP), integral membrane protein 2B (ITM2B/Bri2), artificial intelligence, protein folding, intermolecular interaction, protein processing regulation

## Abstract

Myelin basic protein (MBP) is the second most abundant protein in the central nervous system and is responsible for structural maintenance of the myelin sheath covering axons. Previously, we showed that MBP has a more proactive role in the oligodendrocyte homeostasis, interacting with membrane-associated proteins, including integral membrane protein 2B (ITM2B or Bri2) that is associated with familial dementias. Here, we report that the molecular dynamics of the in silico-generated MBP-Bri2 complex revealed that MBP covers a significant portion of the Bri2 ectodomain, assumingly trapping the furin cleavage site, while the surface of the BRICHOS domain, which is responsible for the multimerization and activation of the Bri2 high-molecular-weight oligomer chaperone function, remains unmasked. These observations were supported by the co-expression of MBP with Bri2, its mature form, and disease-associated mutants, which showed that in mammalian cells, MBP indeed modulates the post-translational processing of Bri2 by restriction of the furin-catalyzed release of its C-terminal peptide. Moreover, we showed that the co-expression of MBP and Bri2 also leads to an altered cellular localization of Bri2, restricting its membrane trafficking independently of the MBP-mediated suppression of the Bri2 C-terminal peptide release. Further investigations should elucidate if these observations have physiological meaning in terms of Bri2 as a MBP chaperone activated by the MBP-dependent postponement of Bri2 membrane trafficking.

## 1. Introduction

The release of amyloidogenic β-amyloid (Aβ) as a result of the β-amyloid precursor protein (APP) proteolytic processing causes Alzheimer’s Disease (AD) [1], which belongs to the group of human amyloid diseases [2]. According to the amyloid cascade hypothesis, the pathological changes in AD arise from the excessive accumulation of Aβ [3,4]. Onset AD is enhanced by mutations thereby increasing Aβ production, especially aggregation-prone Aβ42 [4]. For early- and late-onset sporadic AD cases, Aβ was shown as a major risk factor [5].

At the molecular level, cells have their own mechanisms for maintaining protein homeostasis and controlling of incorrectly folded polypeptides where molecular chaperones play a critical role in maintaining cellular protein homeostasis [6]. These cellular chaperones include Integral Membrane Protein 2B (ITM2B or Bri2) [7]. Supposedly, Bri2 regulates APP processing by masking α- and β-secretase cleavage sites of APP [8,9,10].

The Bri2 protein belongs to the family of integral type II transmembrane domain proteins, and its full-length form consists of 266 aa [7]. The cleavage of the peptide bond between arginine 243 and glutamine 244 residues by pro-protein convertases (PPCs) releases the 23-amino-acid peptide (Bri23) from the C-terminus of Bri2 [11,12]. The extracellular ectodomain of Bri2 also contains the BRICHOS domain, which was initially discovered by sequence similarities with chondromodulin-I and prosurfactant protein C and proposed to be an intramolecular chaperone in the protein folding [13]. The BRICHOS domain of Bri2 is also cleaved by sheddase ADAM10 and released [14]. As for full-length Bri2, BRICHOS has been shown to suppress Aβ42 toxicity by preventing elongation and secondary nucleation in Aβ42 aggregation process [15,16,17]. The intravenous injection of recombinant BRICHOS in Alzheimer’s Disease mouse models attenuated Aβ pathology in the brain and markedly reduced Aβ plaque deposition and the activation of astrocytes and microglia [18]. It was reported that Bri23 also inhibits Aβ42 deposition in vivo [19]. The remaining portion of Bri2, membrane-associated N-terminal fragment (NTF), undergoes intramembrane proteolysis mediated by protease SPPL2a or SPPL2b causing release of the intracellular domain (ICD) into the cytosol and secretion of the remaining TM domain [14].

At the same time, Bri2 itself (particularly Bri23) appears to be a frequent target of disease-associated mutations, including those causing familial British and Danish dementias [20,21] as well as mutations causing familial Chinese and Korean dementias [22,23]. In some studies, Bri23 is called an aggregation-prone region [24] due to amyloidogenic features of its mutated variants ABri and ADan.

Bri2 was recently found as an interactor of myelin basic protein (MBP) [25]. MBP was identified in the early 1970s as the predominant protein of basic protein materials extracted from the whole brain or spinal cord causing experimental allergic encephalomyelitis [26]. In the 1980s, antibodies to MBP were shown to be a hallmark of multiple sclerosis (MS) [27,28,29]. Another insight into the key role of MBP in the formation and compactization of the myelin sheath was a study of shiverer phenotype mice carrying a mutation in the gene encoding MBP [30,31]. MBP is an important structural protein in the central nervous system, where it maintains the dense multilayer assembly of the myelin sheath by adhesion of the opposing cytoplasmic leaflets of the oligodendrocyte membrane [32,33]. Along with this, MBP is an intrinsically disordered protein, which provides its multifunctionality [34]. MBP was reported as a potent inhibitor of Aβ fibrillar assembly [35,36]. The N-terminal 64 amino acids of MBP are responsible for this activity [37].

Previously, using the yeast two-hybrid (Y2H) system, we observed interaction between MBP and Bri23 [25]. The existence of the MBP-Bri23 complex was confirmed in a reciprocal Y2H assay, and putative complexes of MBP with the β-hairpin structure of Bri23 were modeled using a combination of an AI-based AlfaFold2 protein structure modeling service [38] and a High Ambiguity Driven protein–protein *DOCKing* (HADDOCK) [25]. Because MBP was also found among proteins pulled down with Bri2 from the cerebral cortex [39], we hypothesized that the interaction between MBP and Bri2 was supposed to be physiologically relevant. Here, to clarify the details of the intermolecular interaction between MBP and Bri2, we focused on the following questions: Which form of Bri2 does MBP interact with and how does this interaction affect the intracellular behavior of the Bri2 molecule? Our study contained both in silico modeling of the MBP-Bri2 interaction and experimental elucidation how MBP and Bri2 co-expression affects processing and intracellular localization of Bri2, mBri2 (Bri2 without Bri23 C-terminal peptide), and disease-associated Bri2 variants in HEK 293 cells. Our experimental data indicate that MBP attenuates furin-mediated Bri2 cleavage and delays its membrane trafficking, while computational studies provide a structural explanation and support for the observed phenomena.

## 2. Results and Discussion

### 2.1. Molecular Modeling of Individual Proteins Using AlphaFold2

**Figure 1:**
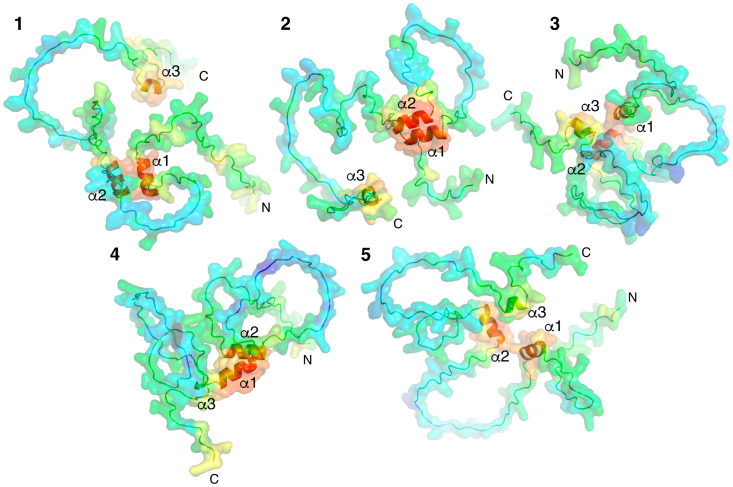
Five AlphaFold2-built 3D models of MBP colored according to their confidence level. pLDDT > 90 (red), pLDDT > 80 (orange), pLDDT > 70 (yellow), pLDDT > 60 (green), pLDDT > 50 (azur), pLDDT > 40 (blue), and pLDDT > 30 (dark blue). Models are numbered (1–5) from maximum to minimum confidence level. Three α-helices and N- and C-termini are indicated.

Models of individual proteins were obtained using an AI-based AlfaFold2 colab service [38]. Five variants of spatial structures of MBP isoform 5 (171 amino acid residues) were generated (Figure 1). MBP is a classical representative of intrinsic disordered proteins (IDPs); therefore, as anticipated, AlphaFold2 predicted five variants of the folding of unstructured regions connecting three short alpha helices, modeled with high confidence.

**Figure 2:**
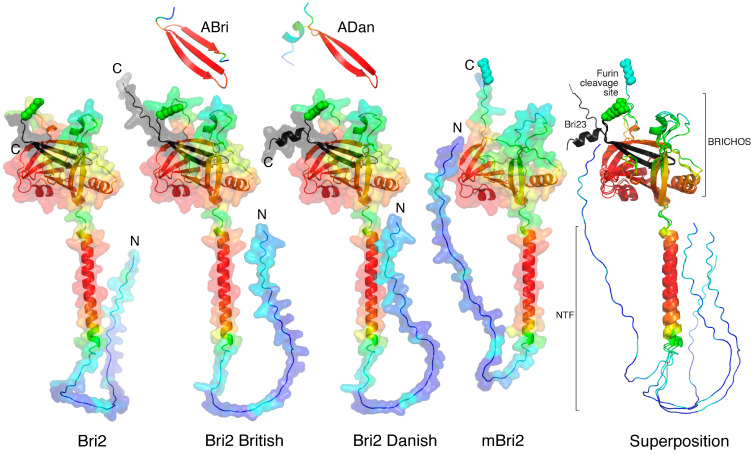
AlphaFold2-built structures of Bri2, mBri2, disease-associated British and Danish Bri2 variants, and corresponding amyloidogenic peptides. The best models are colored according to their confidence levels: pLDDT > 90 (red), pLDDT > 80 (orange), pLDDT > 70 (yellow), pLDDT > 60 (green), pLDDT > 50 (azur), pLDDT > 40 (blue), and pLDDT > 30 (dark blue). The Arg 244 residues at position P1 in furin cleavage site are shown in spheres, and the Bri23 β-hairpin or its mutated variants are shown in black. N and C indicate the corresponding termini of proteins. The BRICHOS domain and the N-terminal fragment (NTF), which includes the ICD and TM of Bri2, are shown on the right as an overlay of the above structures.

As mentioned above, Bri2 consists of three domains. Newly synthesized immature Bri2 consists of an N-terminal unstructured intracellular domain (Bri2 ICD, residues 1–54), a transmembrane region (TM, residues 55–75), which is part of a longer alpha helix, and a C-terminal extracellular ectodomain (residues 76–266). In turn, the Bri2 ectodomain consists of the BRICHOS domain [13,24] and the C-terminal Bri23 peptide, which is cleaved off by furin in the trans-Golgi cisternae of the cell during the transport of Bri2 to the cytoplasmic membrane. As a result, mature Bri2 (mBri2, residues 1–243) is embedded into the plasma membrane without peptide Bri23 [14,19]. During the maturation of disease-associated derivatives of Bri2 (British and Danish), the amyloidogenic peptides ABri and ADan are released instead of Bri23. Figure 2 illustrates the structural roles of the C-terminal peptides (Bri23, Abri, and Adan) in the folding of the corresponding ectodomains.

In all variants, similar three-domain structures are observed with unstructured ICD, long α-helices, and well-structured ectodomains predicted with high confidence (Figure 2). In wild-type Bri2, the British and Danish variant furin cleavage sites are located on a solvent-exposed loop, which separates the BRICHOS domain from the C-terminal β-hairpin. In immature protein, this β-hairpin plays an important role in the formation of the closed structure of the ectodomain, in which the flattened arrangement of the central β-sheet of the BRICHOS domain [24] is covered with a small β-sheet. In addition to the Bri23 β-hairpin, this small β-sheet involves the complementary β-strand, formed from an unstructured region connecting two α-helices localized on both sides of the central β-sheet of the BRICHOS domain (Figure 2), whereas in the mBri23 structure, we can observe the classical solvent-exposed BRICHOS domain.

Disease-associated mutations in the C-terminal region of Bri2 did not affect the folding of the respective ectodomains. Extended C-termini were simply exposed to a solvent and did not participate in intramolecular interactions with other regions of the proteins. Modeling of the secreted C-terminal peptides (ABri and ADan) cleaved from these mutants showed that in the British variant, the mutation in Bri23 leads to the conversion of the β-hairpin into a 3-stranded β-sheet, whereas in the Danish variant, a short α-helix is simply added to the β-hairpin. However, in the latter case, the low reliability of the modeling indicates the possibility of an alternative folding of the peptide.

### 2.2. Simulation of the Intermolecular Interactions during Co-Folding of Two Proteins Using AlphaFold-Multimer

The ITM2 proteins are characterized by extracellular localization of the C-terminal domain. In this case, integration into the membrane, combined with protein biosynthesis on ribosomes assembled into polysomes on the cisterns of the endoplasmic reticulum (ER), does not occur. The protein is synthesized and folded in the cytoplasm, where it exists in a complex with other proteins that perform chaperone and transport functions [40]. At the same time, misfolded and mislocalized proteins are directed for ubiquitination and degradation via the proteasomal machinery [41]. Further integration of ITM2s into the ER membrane also occurs with the participation of a special protein apparatus [40]. And only then, in the Golgi apparatus of the cell, does Bri2 maturation begin, the first stage of which is the cleavage of the Bri23 peptide.

**Figure 3:**
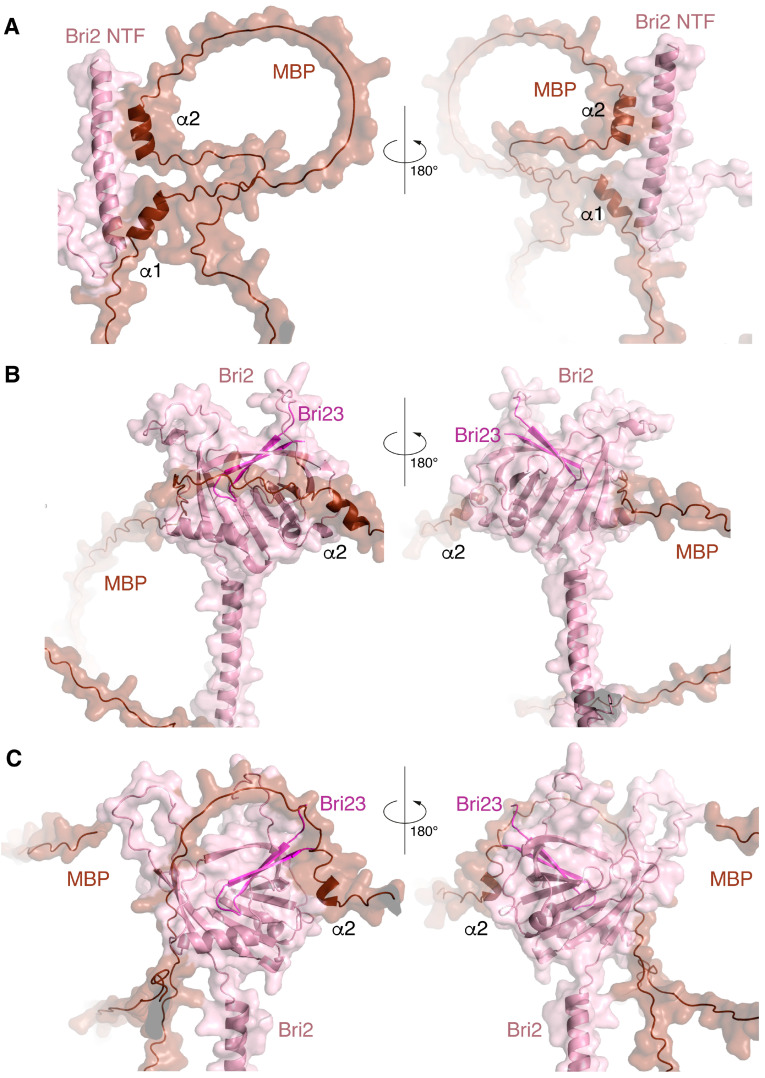
Three complexes with the best interface characteristics obtained by co-folding MBP with various Bri2 fragments using the program AlphaFold-Multimer: the MBP-Bri2 NTF complex 2 (**A**). MBP-Bri2 complex 2 (**B**). MBP-Bri2 complex 3 (**C**). Numbering is given according to Table 1. Two projections (rotated 180 degrees) are shown for each complex. All complexes are prepared in the form of surface cartoon pictograms, where the Bri2 part is colored pink, while Bri23 C-terminal peptide is in magenta, and the MBP part is brown.

The biosynthesis of MBP in the cell is characterized as a localized translation that occurs in the cytoplasm [42]. Previously, using TurboID proximity labeling, we demonstrated the colocalization of MBP with components of ER membrane-targeting SRP machinery including calmodulin, which functions as a chaperon for the ER secretory pathway [43]. A number of components of the proteasomal protein degradation system have also been identified among the cellular partners of MBP [44]. The similar protein environments of MBP and Bri2 during their biosynthesis allowed us to assume the possibility of colocalization of the newly synthesized polypeptides in the cell. We thus aimed to model the process of the co-folding of MBP and Bri2 using the AlphaFold-Multimer 2.3.2, which is an extension of AlphaFold2 that has been specifically built to predict protein–protein complexes [45]. The simulation was carried out using full-length MBP and three variants of Bri2: Bri1-51 (representing Bri2 ICD), Bri1-80 (representing Bri2 NTF), and full-length immature Bri2. Five protein–protein complexes, provided by AlphaFold-Multimer in each case, were analyzed using PDBePisa (Table 1), and the best complexes were visualized by PyMol (Figure 3).

The best structural and energetic parameters were obtained for the MBP complex with full-size Bri2 (model 3 in Table 1); next was the MBP complex with Bri2 NTF (model 2 in Table 1), and with a minimal margin, there was another model MBP-Bri2 (model 2 in Table 1). An analysis of the MBP-Bri2 NTF complex model showed that the main contribution to the formation of the intermolecular interface was provided by the interaction of the alpha–helical regions of both proteins, and no signs of increased structuring and compaction of MBP, expected for the IDPs in the protein–protein complex, were observed (Figure 3).

In the case of the MBP-Bri2 complexes, Bri2 did not undergo significant changes as compared to the structure obtained for the individual protein using AlphaFold2. At the same time, an intermolecular interaction with the well-structured Bri2 ectodomain during co-folding was associated with an increasing disorder in the MBP structure, which manifested in a decrease in the number α-helices and less compaction of the entire molecule (Figure 3). An analysis of intermolecular polar contacts formed in the optimal MBP-Bri2 complexes (Table 2) shows that the residues of the Bri2 ectodomain mainly participate in the interactions. Taken together, the results of the three co-folding experiments indicate that MBP preferentially interacts with the Bri2 ectodomain, which was selected for further protein–protein docking experiments.

### 2.3. Protein–Protein Docking and MD Simulation of the MBP-Bri2Δ64 Complexes

HADDOCK 2.4 [46] was used for further modeling of putative MBP-Bri2 complexes. Because the AlphaFold-Multimer-based experiments indicated that the Bri2 ectodomain is the most probable target for MBP binding, docking and subsequent MD experiments were performed using the Bri2 structure with deleted ICD and half of the TM domain (*Bri2Δ64*).

Three runs of docking were conducted with different Ambiguous Interaction Restraints (AIRs) specified on the surface of the Bri2 ectodomain. All five variants of the MBP and Bri2 structures proposed by AlphaFold2 were used in docking in pairs. Thus, at the final stage of each docking run, we obtained 150 structures (30 structures for each pair of structural models). Lists of the MBP-Bri2*Δ64* complexes obtained in the three docking experiments with characteristics of their intermolecular interfaces are provided in the Supplementary Materials. Table S1 corresponds to the experiment in which the residues of the entire solvent-accessible surface of the Bri2 ectodomain were chosen as AIR (No AIR). Table S2 corresponds to the experiment in which solvent-accessible residues of the C-terminal β-hairpin were chosen as AIR (Bri23-AIR). Table S3 corresponds to the experiment in which all superficial acidic residues of the Bri2 ectodomain were chosen as AIR (acidic AIR). The latter option was due to the presence of extended positively charged clusters on the MBP surface [47].

The analysis of the intermolecular interfaces of the obtained complexes showed that the majority of energy-efficient structures were obtained by the docking experiment with MBP models #3 and #5 (Figure 1) and acidic AIR (for example, complexes 5–6 from Table 3). Though the structure with the largest buried surface area was obtained by the docking experiment with Bri23-AIR and the MBP model 1 (complexes 1–25 from Table 3), the experiment without specifying AIR was the least effective. The characteristics of the two best MBP-Bri2*Δ64* complexes (1–25 and 5–6), which were selected according to the structural and energy characteristics of the intermolecular interfaces provided by the Haddock-based analysis, are summarized in Table 3.

**Figure 4:**
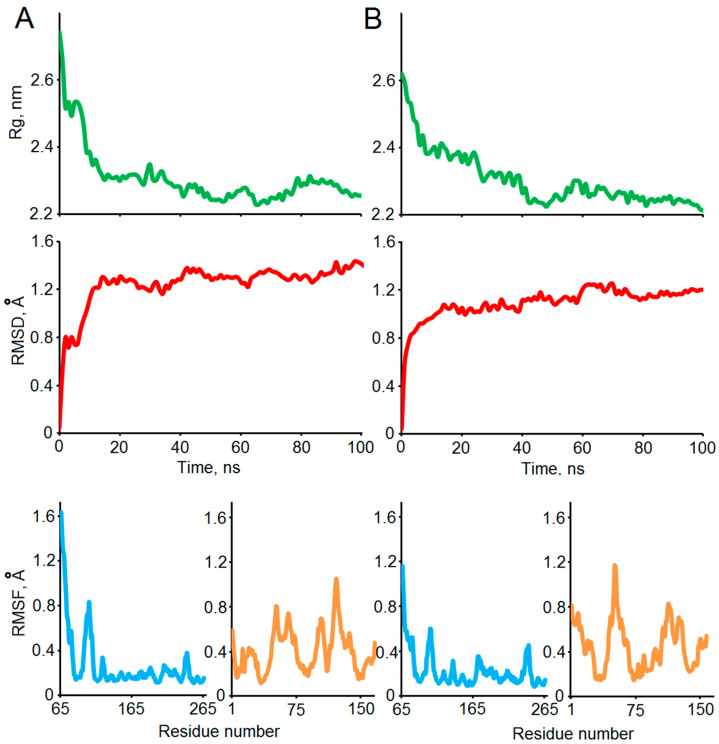
The stabilities of the selected structures 1–25 (**A**) and 5–6 (**B**) of the modeled MBP-Bri2*Δ64* complexes were studied using the 100 ns MD simulation. Time development of the RMSD and Rg throughout the MD trajectory relative to the HADDOCK-built models confirms stabilization of the systems. Residual RMSF values are shown in azur and orange for Bri2 and MBP, respectively.

**Figure 5:**
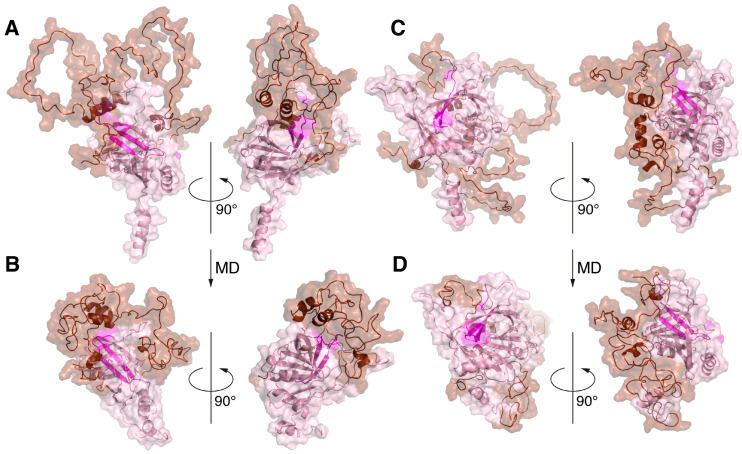
Molecular dynamics simulation of MBP-Bri2*Δ64* complexes results in significant increases in the intermolecular interactions and compacting of the complexes. Two HADDOCK-generated MBP-Bri2 complexes with the best intermolecular interface characteristics: 1–25 (**A**,**B**) and 5–6 (**C**,**D**) before (**A**,**C**) and after (**B**,**D**) 100 ns MD simulations. For both experiments, only part of Bri2 molecule (without ITC and with partial TM domain) was used. Before and after MD complexes are shown in two projections (rotated 90 degrees) in the form of surface cartoon pictograms. The Bri2 part is colored pink; the Bri23 peptide is shown in magenta; and the MBP part is colored brown. N- and C-termini of proteins as well as α-helices of MBP are annotated.

The structural stabilities of the two selected complexes (1–25 and 5–6) obtained in the docking experiments were confirmed by a 100 ns MD simulation. Several standard indicators were calculated to verify the qualities of the MD experiments and affinities of the modeled complexes (Figure 4). According to the backbone root mean square deviation (RMSD), the system was stabilized around 20 ns of the simulation in both cases. The radius of gyration (Rg) values dropped from 2.6 to 2.8 nm to the level of its stabilization around 2.3 nm, which indicates significant compaction of the complexes. The residual root mean square fluctuation (RMSF) levels allow differentiating stable and flexible areas of the interacting proteins, which were further analyzed using a visual inspection of the structures of the complexes before and after MD (Figure 5).

Figure 5 shows significant structural changes in the transition region between the TM and the ectodomain of Bri2, namely changes in the angle between the domains and partial unwinding of the TM α-helix. The structural stabilities of the ectodomain and the α-helical regions of MBP were preserved. The significant compaction of the complexes appeared to be the most interesting result of the MD studies (Figure 4 and Figure 5). A comparative analysis of the intermolecular interfaces provided in Table 4 confirmed that this compaction is indeed associated with the bringing together of previously distant parts of interacting molecules with a corresponding significant increase in the buried surface areas and the number of intermolecular contacts.

As a result, the MBP polypeptide chain covers a significant portion of the Bri2 ectodomain. Thus, there is a high probability that MBP masks the region of the Bri2 surface where the furin cleavage site is located.

**Figure 6:**
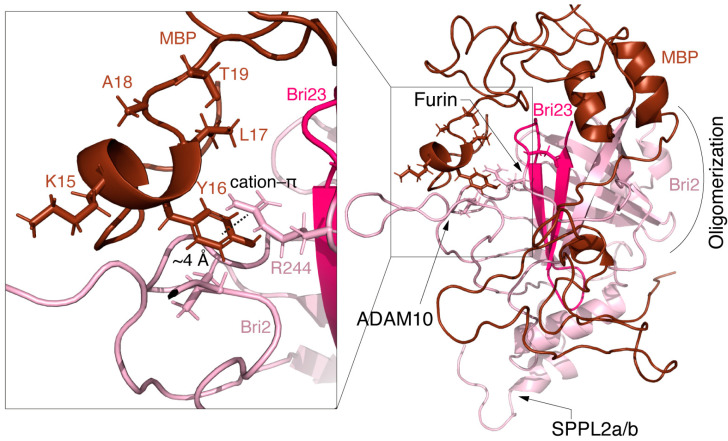
Putative structure of MBP-Bri2 complex obtained by the combination of the protein–protein docking and molecular dynamics. MBP is colored brown; Bri2 is colored salmon; and Bri23 peptide is colored pink. Proteolytic processing sites are shown by arrows in the Bri2 structure. Insert shows hydrophobic cavity stabilized by Bri2 R244–MBP Y16 cation–π interaction.

Figure 6 shows the details of the MBP-Bri2 interactions in the MBP-Bri2 complex (1–25) discussed above. One can see that the N-terminal part of MBP masks the P1 amino acid residue of the furin cleavage site in a hydrophobic cavity stabilized by the cation–π interaction of Bri2 Arg244 with MBP Tyr16, while the ADAM10 and SPPL2a/b cleavage sites remain available for proteolysis.

The data obtained in silico encouraged us to study the effect of MBP on processing and cellular localization of full-length Bri2, mBri2, and its disease-associated variants.

### 2.4. MBP Modulates Post-Translational Bri2 Processing and Affects Its Intracellular Localization by Restriction of Furin-Catalyzed Release of Its C-Terminal Peptide

To analyze the interaction of Bri2 and MBP in mammalian cells, we generated a series of DNA constructs to express full-length human Bri2 (Bri2), Bri2 with deletion of the Bri23 peptide (mBri2), and Bri2 variants carrying either a mutation at the stop codon (British [20], Bri2 Bri) or 10-nucleotide duplication of the one codon before the normal stop codon (Danish [21], Bri2 Dan). Two additional expression constructs were prepared for the protein C-terminally flanked with an HA epitope: one for Bri2 and another for Bri2 with alanine substitutions (K243A and R244A) in the furin cleavage site (Bri2-HA and Bri2 KRmut, respectively). Further details for the constructs used in the study are provided in Supplementary Figure S1. All Bri2 variants contained the N-terminal Strep-Tag.

In order to probe the interplay between Bri2 and MBP, we used the HEK 293T cell line. Usage of non-myelin-forming cells is an evident limitation of our study, which may result in the loss of oligodendrocyte-specific pathways and interactors. Nonetheless, here we would like to note three major points for the utilization of HEK 293T. Firstly, Bri2 mRNA was found highly expressed in the brain, placenta, pancreas, and kidney [20]; thus, it may be suggested that basic the mechanism and regulation of its processing are shared between HEK 293T cells and oligodendrocytes. Secondly, Schwann cells and oligodendrocytes already are overexpressing MBP. Therefore, the only way to show the effect of MBP on Bri2 processing is to suppress MBP expression by siRNA, knockout of MBP gene, or epigenetic regulation. Systemic cell stress caused by MBP downregulation in these cells will unpredictably affect experimental outcomes. Thirdly, according to a study by Barbarese and Pfeiffer [49], oligodendrocytes can accumulate MBP at the rate of 0.2 fmol per day per oligodendrocyte and reach a dynamic equilibrium of 1 fmol of MBP per cell. Together with the data reporting that the volume of mature oligodendrocytes is approximately 1.7 pL [50] and assuming that MBP is uniformly distributed in the cell, the concentration of MBP inside an oligodendrocyte is 0.6 mM. If we consider that only 10% of this total MBP is “free” and that the remainder is membrane-associated [49], this concentration is abundantly more or at a minimum equal to the transient transfection of HEK 293T cells by the MBP-coding vector.

**Figure 7:**
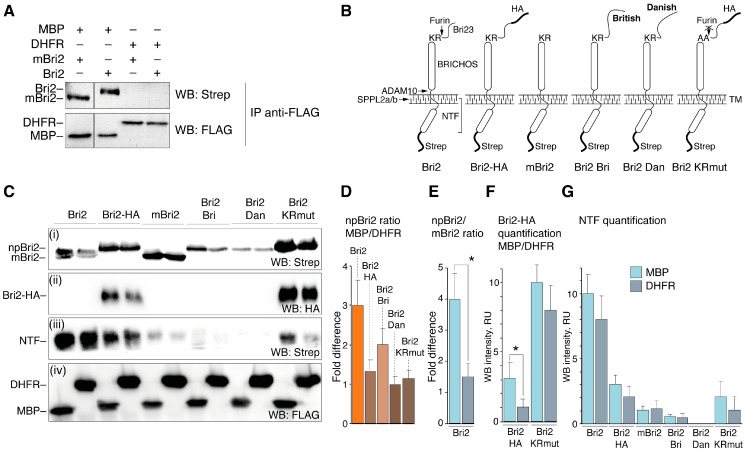
MBP interacts with Bri2 and modulates its intracellular processing. (**A**) Immunoprecipitation (IP) analysis demonstrating the Bri2-MBP interaction. The IP eluates were separated by SDS-PAGE, transferred to the nitrocellulose membrane, and immunoblotted with indicated antibodies. The plasmids used in transfection are shown on the top. The antibodies used in Western blotting are shown on the right. (**B**) Schematic representation of Bri2 variants used in the co-expression experiments. (**C**) Immunoblot of whole-cell lysates (WCLs) of HEK293T cells overexpressing Bri2 variants with MBP (left) or DHFR (right). (**D**) Quantification of the ratio of non-processed Bri2 (npBri2) in MBP and DHFR co-expressing cells related to WB panel **C**(**i**). (**E**) Quantification of the ratio of npBri2 and its processed form mBri2 related to WB panel (**C**(**i**)). (**F**) Comparison of the protein level of HA-tagged Bri2 and Bri2 KRmut in MBP co-expressing cells and DHFR co-expressing cells related to WB panel (**C**(**ii**)). (**G**) Comparison of the NTF release in MBP co-expressing cells and DHFR co-expressing cells related to WB panel (**C**(**iii**)). In (**E**–**G**), light blue and grey bars indicate MBP and DHFR co-expression, respectively. Average values with standard deviations are shown. Statistically significant difference is marked by asterisk.

At first, the HEK 293T cells were co-transfected with either Bri2 or mBri2 constructs together with plasmids coding for FLAG-tagged MBP or dihydrofolate reductase (DHFR) as a control. An immunoprecipitation analysis (Figure 7A) demonstrated that MBP indeed interacts with both unprocessed Bri2 and mBri2.

Next, all generated Bri2 variants (Figure 7B) were used for a similar co-transfection experiment followed by immunoblot analysis of the whole-cell lysates (Figure 7C). Western blot analysis revealed that protein bands corresponding to two forms of Bri2: full-length non-processed Bri2 (npBri2) and mBri2 were clearly observed only in the case of transfection with wild-type Bri2 (Figure 7C(i)). The ratio of npBri2 in MBP-co-expressing cells to npBri2 in DHFR-co-expressing cells (npBri2 ratio MBP/DHFR) increased in cases of wild-type Bri2 and its British form (Bri2 Bri) (Figure 7D). Moreover, co-expression of wild-type Bri2 with MBP significantly increased the npBri2/mBri2 ratio compared with the control transfection (Figure 7E). Monitoring of the protein expression using an HA epitope (Bri2-HA quantification MBP/DHFR) showed a similar increase in the npBri2 level during co-expression with MBP (Figure 7F). Mutation of the furin cleavage site in Bri2 KRmut resulted in a dramatic increase in unprocessed Bri2 regardless of the co-expressed protein (Figure 7F). Quantification of the NTF release in various co-transfections (Figure 7G) suggests that co-expression of the Bri2 variants with MBP or DHFR has no significant effect on the NTF cleavage, whereas any mutations suppress this process regardless of the type of co-expressed protein.

To summarize, our data indicate that MBP indeed suppresses furin-mediated processing but does not affect the NTF release catalyzed by ADAM10 and SPPL2a/b.

Next, we analyzed the effect of MBP on the subcellular localization of Bri2. To exclude the possibility that the MBP-mediated suppression of the Bri2 cleavage by furin affects the distribution of Bri2, we used the Bri2 KRmut variant resistant to furin hydrolysis.

**Figure 8:**
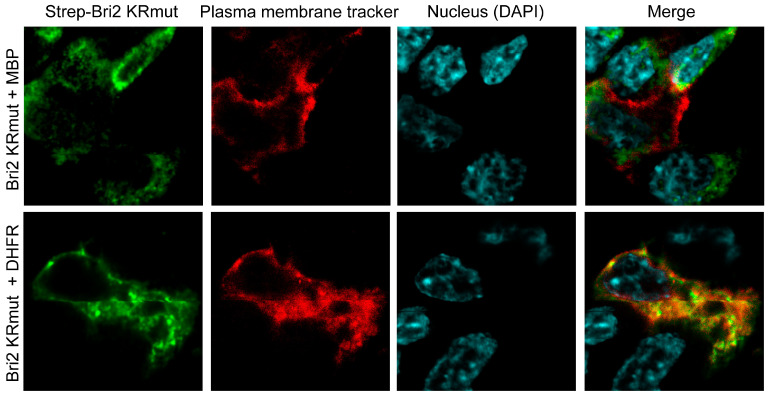
Immunofluorescence staining of overexpressed Bri2 KRmut in the presence of MBP or DHFR in HEK 293T cells. Mouse anti-Strep antibodies (BioRad, Hercules, CA, USA) and DyLight649-labeled secondary goat anti-mouse IgG antibodies (Rockland Immunochemicals, Limerick, PA, USA) were used to stain Bri2 proteins. The expressed proteins are annotated on the left.

HEK 293T cells were transfected with plasmids coding for Bri2 KRmut and red fluorescent plasma membrane tracker together with plasmids expressing either MBP or DHFR. Twenty-four hours after transfection, the cells were seeded on laminin-treated glass slides, and on the next day, the cells were washed and fixed in formaldehyde. Further, cells were stained with anti-Strep primary antibodies followed by the staining with respective fluorescent dye-labeled secondary antibodies, whereas nuclei were contrasted with DAPI. The images were captured using a confocal laser scanning microscope focused on the broadest cell section exposing the nucleus and membrane (Figure 8).

Our data reveal that in the presence of DHFR (control), the Bri2 KRmut protein was readily colocalized with the plasma membrane (Figure 8, lower row). In contrast, we showed that the MBP-Bri2 co-expression altered the Bri2 KRmut localization, significantly sequestering its membrane staining (Figure 8, upper row). Thus, MBP restricts Bri2 membrane trafficking independently of the suppression of the furin-catalyzed C-terminal Bri23 peptide release.

## 3. Materials and Methods

### 3.1. Plasmids Coding for Bri2 Variants and MBP

Nucleotide sequences coding for full-length Bri2 and mBri2 were amplified using cDNA isolated from HEK293T cells, then cloned to pcDNA3-N-Strep plasmid at *HindIII* and *EcoRI* sites. Bri2 variants were generated by site-directed PCR mutagenesis using pcDNA3N-Strep-Bri2 plasmid as a template with two multidirectional 5′-overlapping primers carrying nucleotide substitutions: British variant (5′-caagaaaaacattattgaggaaaattaagaattctgcagatatccatcacactggc-3′ and 5′-ctcaataatgtttttcttgactgttctagaacaaattaaagtttccacggcaaatttg-3′) and Danish variant (5′–gttcttgaacagtcaagaaaaacattattgagaattctgcagatatccatcacactggc-3′ and 5′–gactgttcaagaacaaattaaaacaaattaaagtttccacggcaaatttg-3′) and Bri2 variant with C-terminal HA tag (5′–gatgttccagattacgcttaagaattctgcagatatccatcacactggcg-3′ and 5′–gtaatctggaacatcgtatgggtaagaacaaattaaagtttccacgg-3′).

Bri2 KRmut with K243A and R244A substitutions in the furin cleavage site was generated similarly by site-directed PCR mutagenesis using pcDNA3-N-Strep-Bri2-HA plasmid as a template with two multidirectional 5′-overlapping primers (5′–caggctgccgaagccagcaattgtttcgcaattcg-3′ and 5′–ttcggcagcctgaatacctttaatagtttctctgcgttgcag-3′).

Preparation of pcDNA3 plasmids coding for MBP or DHFR N-terminal flanked with FLAG epitope were described in [51].

### 3.2. Transient Transfection of Mammalian Cells

HEK 293T cells were obtained from the Russian Cell Culture Collection (RCCC, Institute of cytology of the Russian Academy of Sciences). HEK 293T cells were maintained by passage in Dulbecco’s modified Eagle’s medium supplemented with 100 μg/mL streptomycin, 100 units/mL penicillin, and 10% fetal bovine serum (FBS) (pH 7.2–7.4) in a humidified atmosphere containing 5% CO_2_ at 37 °C. HEK293T cells were transfected with pcDNA3 plasmids coding for N-terminal Strep-Tag Bri2 variants (Bri2, mBri2, Bri2 Bri, and Bri2 Dan) and N-terminal Strep plus C-terminal HA-tagged Bri2 variants (Bri2-HA and Bri2 KRmut) together with pcDNA3 plasmids coding for either MBP or DHFR N-terminal flanked with FLAG epitope [51] using Lipofectamine LTX Reagent with PLUS Reagent (Thermo Fisher Scientific, Waltham, MA, USA) following the manufacturer’s instructions. All the experiments were conducted at 48 h after transfection.

### 3.3. Antibodies

Monoclonal ANTI-FLAG M2-Peroxidase (HRP) antibody produced in mouse (Merck, Rahway, NJ, USA), monoclonal ANTI-FLAG M2 antibody produced in mouse (Merck), mouse anti Strep-Tag Classic antibody, clone Strep-Tag II purified (BioRad), mouse anti Strep-Tag Classic antibody, clone Strep-Tag II HRP conjugated (BioRad), and goat polyclonal anti-mouse IgG (H&L) Antibody DyLight 649 Conjugated Pre-Adsorbed (Rockland Immunochemicals) were used.

### 3.4. Immunoprecipitation and Immunoblotting

Forty-eight hours after transfection, cells were washed with ice-cold PBS and lysed in 1xTNE buffer (50 mm Tris–HCl (pH 7.4), 100 mm NaCl, and 1 mM EDTA) containing 1% octyl beta-D-glucopyranoside, 1mM phenylmethane sulfonyl fluoride, and Protease Inhibitor Cocktail (Merck) for 30 min on ice. Collected lysates were sonicated, and debris was removed by centrifugation at 10,000 *g* for 20 min. The cleared lysates were incubated with 20 μL of ANTI-FLAG M2 Affinity Gel (Merck) slurry at 4 °C for overnight. After incubation, agarose beads with immunocomplexes were washed with TNE buffer five times, and immunocomplexes were eluted from agarose beads with sample buffer (SB, 65.8 mM Tris·HCl, pH 6.8, 10% glycerol, 1% SDS, and 0,01% bromophenol blue) at 65 °C for 5 min. The supernatants were treated with 5 μL of 2-mercaptoethanol at 65 °C for 5 min. Supernatants containing immunocomplexes along with corresponding whole-cell lysate samples prepared from cleared lysates and SB were resolved by SDS-PAGE, transferred to the nitrocellulose membrane, and immunoblotted with appropriate antibodies.

### 3.5. Immunofluorescence, Image Acquisition, and Analysis in Cell Culture

HEK293T cells were transfected with plasmids coding for Bri2 KRmut and membrane tracker fused with red fluorescent protein (pFusionRed-f-mem, Evrogen, Moscow, Russia) together with plasmids coding for MBP or DHFR. Next day, cells were seeded on laminin-coated coverslips and fixed on the next day (48 h in total) with the 4% solution of formaldehyde in PBS for 20 min. The reaction was stopped by treatment 3 × 5 min with 1 M Gly in PBS, pH 8.5. Cells were permeabilized and blocked with 0.1% saponin and 5% normal goat serum in PBS for 1 h at room temperature (RT). Cells were incubated with primary anti-Strep antibodies (1:200) in PBS with 0.1% saponin and 1% BSA overnight at 4 °C. Slides were washed in PBS 3 × 5 min at room temperature then incubated with secondary goat anti-mouse IgG (H&L) DyLight 649 Conjugated Antibody (1:1000) for 2 h at RT. Nuclei were counterstained with 2.5 μM 4′,6-diamidino-2-phenylindole, and the coverslips were mounted on glass slides using ProLong Glass Antifade Mountant (Invitrogen, P36980). Images were captured on an FV10i FluoView microscope (Olympus, Tokyo, Japan) using a 60× objective (with a zoom of 4.5, 270× magnification in total) with aperture size of 1 enabling high-quality imaging.

### 3.6. AlphaFold2 and AlphaFold-Multimer Based Modeling

Spatial structures of MBP isoform 5 (UniProt ID P02686-5) and human Bri2 (UniProt ID Q9Y287), as well as their respective mutated variants, were prepared using AlphaFold2 colab service (https://colab.research.google.com/github/deepmind/alphafold/blob/main/notebooks/AlphaFold.ipynb, accessed on 1 July 2023) [38]. All 5 models obtained for each protein were used in the protein–protein docking experiments.

To simulate the formation of a complex during co-expression of two proteins, we used AlphaFold-Multimer 2.3.2 (https://github.com/google-deepmind/alphafold/releases/tag/v2.3.2, accessed on 1 August 2023) [45]. AlphaFold-Multimer version 2.3.2 generates several structures per model by default. The simulation was carried out in three variants using a full-size MBP-5 and three variants of Bri2: Bri1-51 representing a N-terminal intracellular domain of Bri2 (ICD), Bri1-80 representing intracellular and transmembrane domains (NTF), and full-sized Bri2. In each case, all 5 models were used for analysis.

PyMOL Molecular Graphics System (https://pymol.org, accessed on 1 September 2023) was used for both visualization of the obtained structures of individual proteins and complexes and for color-based assessment of the model qualities according to the AlphaFold-predicted local distance difference test (pLDDT), which estimated per-residue confidence on a scale from 0 to 100. Regions with pLDDT > 90 are expected to have very high confidence; regions with pLDDT between 70 and 90 are expected to have good confidence; regions with pLDDT between 50 and 70 are of low confidence, and pLDDT < 50 is a strong predictor of IDPs suggesting that such region is unstructured in physiological conditions.

### 3.7. Molecular Docking

The 3D models of the complexes were obtained with HADDOCK 2.4, (https://www.bonvinlab.org/software/haddock2.4/ accessed on 1 September 2023) [46,52] according to the three-stage algorithm. At the first stage, rigid body docking was performed, during which molecules were rotated and translated randomly to minimize intermolecular energy. At the second stage, semi-flexible refinement (it1) stage, annealing of torsion angle space was performed to refine the orientation of the whole molecules and their residues forming intermolecular interfaces. At the third stage of explicit solvent refinement (water), the structures were refined in explicit solvent layers. The 3D coordinates of the five MBP-5 and Bri2 models generated by AlphaFold2 were used in pairs. In most calculations, AIRs with 40% solvent-accessible surface area were defined. To assess solvent accessibilities of the surface residues, the program FreeSASA was used [53].

At the first stage of docking, 1000 structures of the complexes were calculated. At the second stage, 200 structures with the lowest AIR violations were energy-minimized with the side chains left flexible. At the third stage, the 30 best structures were minimized in the 8 Å shell of explicit TIP3P water [54]. These best structures were grouped into clusters based on the FCC-clustering algorithm [46]. The default scoring function (HADDOCK scores) settings for protein–protein complexes were used: HADDOCK score (it0) = 0.01 Evdw + 1.0 Eelec + 1.0 Edesol + 0.01 Eair − 0.01 BSA, HADDOCK score (it1) = 1.0 Evdw + 1.0 Eelec + 1.0 Edesol + 0.1 Eair − 0.01 BSA, HADDOCK score (water) = 1.0 Evdw + 0.2 Eelec + 1.0 Edesol + 0.1 Eair, where Evd—van der Waals intermolecular energy, Eelec—electrostatic intermolecular energy, Edesol—desolvation energy calculated using the empirical atomic solvation parameters from [48], Eair—distance restraints (AIR) energy, and BSA—buried surface area.

In addition to the HADDOCK score, ranking complexes according to the weighted sum of several parameters, PyMOL, and PDBePisa (https://www.ebi.ac.uk/pdbe/pisa/ accessed on 1 September 2023) were used for analysis and visualization of the interactions.

### 3.8. Molecular Dynamics of Obtained Complexes

Molecular dynamics (MD) simulations were performed using the GROMACS 2020.3 software package [55] with the Amber ff99SB-ILDN force field [56]. Each complex was placed in the center of a cubic simulation box, whose size was chosen to ensure that no less than 1.5 nm distance was between the edge of the box and any protein atom. The box was filled with an explicit solvent using the TIP3P water model [57]. The charge of the system was neutralized by complementing the solvent with Na^+^ and Cl^−^ ions.

The energy of the system was minimized using the steepest descent method for 50,000 steps until the force on any atom dropped below 1000 kJ/(M∙nm^−2^). Then, the temperature and pressure were equilibrated during 100 ps each using the modified Berendsen (V-rescale) [58] and Parrinello–Raman [59] algorithms, respectively. Productive MD simulation was conducted during 100 ns in the same isothermal–isobaric ensemble. The leap-frog algorithm with a time step of 2 fs was used for integration [60]. Hydrogen bonds and long-range electrostatic interactions were maintained using the LINCS algorithm and PME summation method, respectively.

The trajectories were prepared for analysis using the command “*gmx trjconv*” with the flag “*−pbc nojump*”. The energy-minimized structures after the barostat step were assigned as the reference structures of the complexes. Rg (radius of gyration), RMSF, and RMSD were calculated using the commands “*gmx gyrate*”, “*gmx rmsf*”, and “*gmx rms*”, respectively.

## 4. Conclusions

**Figure 9:**
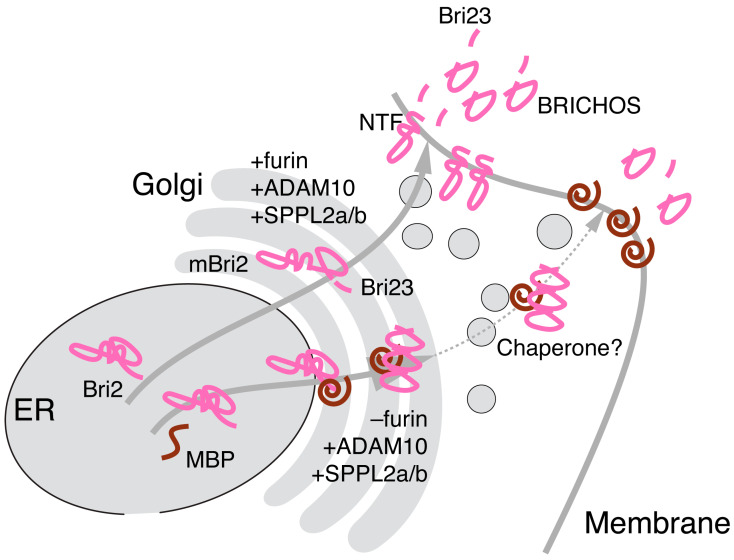
Bri2 as a potential MBP chaperone. Co-expression of MBP and Bri2 results in suppression of furin, but not ADAM10- and SPPL2a/b-mediated Bri2 processing. Multimerization of BRICHOS domains activates Bri2 high-molecular-weight oligomer chaperone function, which provides MBP membrane trafficking. Cellular organelles are presented in grey, Bri2 and its domains are presented in pink, and MBP is presented in brown.

Here, we attempted to simulate the interaction of MBP with Bri2 using a combinatorial computational approach including AlfaFold2- and AlphaFold-Multimer-based modeling in combination with protein–protein docking by HADDOCK and a stability evaluation of the modeled complexes by molecular dynamics. Our in silico experiments indicate a high probability that the MBP molecule masks the furin cleavage site on the surface of Bri2 and provides a structural explanation and support for our experimental findings of MBP-dependent attenuation of furin-mediated Bri2 cleavage and its subsequent transport to the membrane. On this basis, we further speculate that MBP does not interact with the opposite surface of the Bri2 BRICHOS domain, which is responsible for the multimerization and activation of the Bri2 high-molecular-weight oligomer chaperone function [17,61]. To Conclude, we suggest that Bri2 may function as a MBP chaperone activated by the MBP-dependent postponement of Bri2 membrane trafficking, as shown in Figure 9.

## Figures and Tables

**Table 1:** Characteristics of intermolecular interfaces in the five MBP-Bri2 complexes modeled with AlphaFold-Multimer using full-length Bri2 or its N-terminal fragments ICD and NTF.

**MBP-Bri2 ICD Complexes**					
	1	2	3	4	5
Interface area *, Å^2^	552	342.7	484.3	-	-
ΔG **, kcal/mol	−11.0	0.7	1.1	-	-
ΔG, *p*-value	0.235	0.798	0.867	-	-
Hydrogen bonds	0	4	4	-	-
Salt Bridges	0	0	5	-	-
**MBP-Bri2 NTF Complexes**					
	1	2	3	4	5
Interface area *, Å^2^	456.8	906.7	531.3	528.7	62.1
ΔG **, kcal/mol	−10.8	−15.0	−7.2	−10.4	−1.8
ΔG, *p*-value	0.299	0.481	0.503	0.394	0.289
Hydrogen bonds	0	3	4	0	0
Salt Bridges	0	7	1	0	0
**MBP-Bri2 Complexes**					
	1	2	3	4	5
Interface area * Å^2^	820.9	979.6	1718.9	576.3	327.9
ΔG **, kcal/mol	−10.2	−15.0	−22.5	−8.8	−4.0
ΔG, *p*-value	0.372	0.263	0.129	0.288	0.293
Hydrogen bonds	7	2	14	0	0
Salt Bridges	0	3	0	0	0

* Calculated as difference in total accessible surface areas of isolated and interfacing structures divided by two. ** The solvation free energy gain upon formation of the interface calculated as difference in total solvation energies of isolated and interfacing structures. It does not include the effect of satisfied hydrogen bonds and salt bridges across the interface.

**Table 2:** Polar interactions in the intermolecular interfaces of the MBP-Bri2 complexes modeled with AlphaFold-Multimer.

	MBP			Bri2		
N	AA	Atom	Distance Å	AA	Atom	Contact Type *
2	Ser116	N	3.32	Gly90	O	HB
2	Ser116	O	3.08	Gly90	N	HB
2	Arg98	NE	3.27	Glu235	OE1	SB
2	Arg98	NH1	2.53	Glu235	OE1	SB
2	Arg98	NH2	3.28	Glu235	OE2	SB
3	Arg108	NH2	3.15	Asn101	OD1	HB
3	Leu110	N	3.81	Asp96	OD1	HB
3	Leu110	N	3.62	Asp96	OD2	HB
3	Ser111	N	3.43	Asp96	OD1	HB
3	Ser113	N	3.45	Pro194	O	HB
3	Ser113	OG	2.26	Ser196	OG	HB
3	Ser113	OG	3.27	Ser196	O	HB
3	Gly118	N	3.77	Tyr88	O	HB
3	Phe90	O	3.46	Lys230	NZ	HB
3	Ser111	O	3.43	Ser196	N	HB
3	Ser111	O	3.46	Tyr197	N	HB
3	Leu112	O	3.84	Ile94	N	HB
3	Ser113	OG	2.96	Ser196	N	HB
3	Glu120	OE2	3.68	Tyr88	OH	HB

* According to PDBePISA: SB is for salt bridge; HB is for hydrogen bond.

**Table 3:** Haddock-based analysis of intermolecular interfaces of the two best MBP-Bri*Δ64* complexes.

	Intermolecular Energy, kcal/mol					Buried Surface, Å
Model	Total	Vdw ^1^	Elec ^1^	dH ^2^	Edesol ^3^	
1–25	−414.6	−97.8	−316.8	−344.4	−37.3	4042.2
5–6	−473.6	−86.8	−386.8	−352.5	−24.2	3802.7

^1^ Van der Waals (Vdw) and electrostatic (Elec) energy for the intermolecular interaction calculated with HADDOCK. ^2^ Binding energy (Etotal complex − Sum[Etotal components]) ^3^ Empirical desolvation energy [48].

**Table 4:** Characteristics of intermolecular interfaces in the two selected MBP-Bri2*Δ64* complexes before and after MD evaluation (according to PDBePISA).

Complex	1–25 (Figure 5A,B)		5–6 (Figure 5C,D)	
Characteristics/Structures	HADDOCK	MD	HADDOCK	MD
interface area, Å^2^	1921.3	2587.0	1823	3056
ΔG, kcal/mol	−18.9	−15.9	−12.0	−10.2
ΔG, *p*-value	0.263	0.504	0.635	0.585
Hydrogen bonds	15	27	14	44
Salt Bridges	3	10	8	29

## Data Availability

Data are contained within the article and Supplementary Materials.

## Supplementary Materials

The following supporting information can be downloaded at https://www.mdpi.com/article/10.3390/ijms25052608/s1.

## References

[1] Kim D., Tsai L.-H. (2009). Bridging Physiology and Pathology in AD. Cell.

[2] Benson M.D., Buxbaum J.N., Eisenberg D.S., Merlini G., Saraiva M.J.M., Sekijima Y., Sipe J.D., Westermark P. (2018). Amyloid Nomenclature 2018: Recommendations by the International Society of Amyloidosis (ISA) Nomenclature Committee. Amyloid.

[3] Hardy J.A., Higgins G.A. (1992). Alzheimer’s Disease: The Amyloid Cascade Hypothesis. Science.

[4] Hardy J. (2017). The Discovery of Alzheimer-causing Mutations in the APP Gene and the Formulation of the “Amyloid Cascade Hypothesis”. FEBS J..

[5] Kunkle B.W., Grenier-Boley B., Sims R., Bis J.C., Damotte V., Naj A.C., Alzheimer Disease Genetics Consortium (ADGC), The European Alzheimer’s Disease Initiative (EADI), Cohorts for Heart and Aging Research in Genomic Epidemiology Consortium (CHARGE), Genetic and Environmental Risk in AD/Defining Genetic, Polygenic and Environmental Risk for Alzheimer’s Disease Consortium (GERAD/PERADES) (2019). Genetic Meta-Analysis of Diagnosed Alzheimer’s Disease Identifies New Risk Loci and Implicates Aβ, Tau, Immunity and Lipid Processing. Nat. Genet..

[6] Balchin D., Hayer-Hartl M., Hartl F.U. (2020). Recent Advances in Understanding Catalysis of Protein Folding by Molecular Chaperones. FEBS Lett..

[7] Deleersnijder W., Hong G., Cortvrindt R., Poirier C., Tylzanowski P., Pittois K., Van Marck E., Merregaert J. (1996). Isolation of Markers for Chondro-Osteogenic Differentiation Using cDNA Library Subtraction. J. Biol. Chem..

[8] Fotinopoulou A., Tsachaki M., Vlavaki M., Poulopoulos A., Rostagno A., Frangione B., Ghiso J., Efthimiopoulos S. (2005). BRI2 Interacts with Amyloid Precursor Protein (APP) and Regulates Amyloid β (Aβ) Production. J. Biol. Chem..

[9] Matsuda S., Giliberto L., Matsuda Y., Davies P., McGowan E., Pickford F., Ghiso J., Frangione B., D’Adamio L. (2005). The Familial Dementia BRI2 Gene Binds the Alzheimer Gene Amyloid-β Precursor Protein and Inhibits Amyloid-β Production. J. Biol. Chem..

[10] Matsuda S., Giliberto L., Matsuda Y., McGowan E.M., D’Adamio L. (2008). BRI2 Inhibits Amyloid β-Peptide Precursor Protein Processing by Interfering with the Docking of Secretases to the Substrate. J. Neurosci..

[11] Kim S.-H., Wang R., Gordon D.J., Bass J., Steiner D.F., Lynn D.G., Thinakaran G., Meredith S.C., Sisodia S.S. (1999). Furin Mediates Enhanced Production of Fibrillogenic ABri Peptides in Familial British Dementia. Nat. Neurosci..

[12] Kim S.-H., Creemers J.W.M., Chu S., Thinakaran G., Sisodia S.S. (2002). Proteolytic Processing of Familial British Dementia-Associated BRI Variants. J. Biol. Chem..

[13] Sánchez-Pulido L., Devos D., Valencia A. (2002). BRICHOS: A Conserved Domain in Proteins Associated with Dementia, Respiratory Distress and Cancer. Trends Biochem. Sci..

[14] Martin L., Fluhrer R., Reiss K., Kremmer E., Saftig P., Haass C. (2008). Regulated Intramembrane Proteolysis of Bri2 (Itm2b) by ADAM10 and SPPL2a/SPPL2b. J. Biol. Chem..

[15] Arosio P., Michaels T.C.T., Linse S., Månsson C., Emanuelsson C., Presto J., Johansson J., Vendruscolo M., Dobson C.M., Knowles T.P.J. (2016). Kinetic Analysis Reveals the Diversity of Microscopic Mechanisms through Which Molecular Chaperones Suppress Amyloid Formation. Nat. Commun..

[16] Poska H., Haslbeck M., Kurudenkandy F.R., Hermansson E., Chen G., Kostallas G., Abelein A., Biverstål H., Crux S., Fisahn A. (2016). Dementia-Related Bri2 BRICHOS Is a Versatile Molecular Chaperone That Efficiently Inhibits Aβ42 Toxicity in *Drosophila*. Biochem. J..

[17] Chen G., Abelein A., Nilsson H.E., Leppert A., Andrade-Talavera Y., Tambaro S., Hemmingsson L., Roshan F., Landreh M., Biverstål H. (2017). Bri2 BRICHOS Client Specificity and Chaperone Activity Are Governed by Assembly State. Nat. Commun..

[18] Manchanda S., Galan-Acosta L., Abelein A., Tambaro S., Chen G., Nilsson P., Johansson J. (2023). Intravenous Treatment with a Molecular Chaperone Designed against β-Amyloid Toxicity Improves Alzheimer’s Disease Pathology in Mouse Models. Mol. Ther..

[19] Kim J., Miller V.M., Levites Y., West K.J., Zwizinski C.W., Moore B.D., Troendle F.J., Bann M., Verbeeck C., Price R.W. (2008). BRI2 (ITM2b) Inhibits A Deposition In Vivo. J. Neurosci..

[20] Vidal R., Frangione B., Rostagno A., Mead S., Révész T., Plant G., Ghiso J. (1999). A Stop-Codon Mutation in the BRI Gene Associated with Familial British Dementia. Nature.

[21] Vidal R., Révész T., Rostagno A., Kim E., Holton J.L., Bek T., Bojsen-Møller M., Braendgaard H., Plant G., Ghiso J. (2000). A Decamer Duplication in the 3′ Region of the *BRI* Gene Originates an Amyloid Peptide That Is Associated with Dementia in a Danish Kindred. Proc. Natl. Acad. Sci. USA.

[22] Rhyu J.-M., Park J., Shin B.-S., Kim Y.-E., Kim E.-J., Kim K.W., Cho Y.G. (2023). A Novel c.800G>C Variant of the ITM2B Gene in Familial Korean Dementia. JAD.

[23] Liu X., Chen K.-L., Wang Y., Huang Y.-Y., Chen S.-D., Dong Q., Cui M., Yu J.-T. (2021). A Novel ITM2B Mutation Associated with Familial Chinese Dementia. JAD.

[24] Leppert A., Poska H., Landreh M., Abelein A., Chen G., Johansson J. (2023). A New Kid in the Folding Funnel: Molecular Chaperone Activities of the BRICHOS Domain. Protein Sci..

[25] Smirnova E.V., Rakitina T.V., Saratov G.A., Kudriaeva A.A., Belogurov A.A. (2022). Deconvolution of the MBP-Bri2 Interaction by a Yeast Two Hybrid System and Synergy of the AlphaFold2 and High Ambiguity Driven Protein-Protein Docking. Crystals.

[26] Eylar E.H., Salk J., Beveridge G.C., Brown L.V. (1969). Experimental Allergic Encephalomyelitis. Arch. Biochem. Biophys..

[27] Ryberg B. (1978). Multiple Specificities of Antibrain Antibodies in Multiple Sclerosis and Chronic Myelopathy. J. Neurol. Sci..

[28] Panitch H.S. (1980). CSF Antibody to Myelin Basic Protein: Measurement in Patients with Multiple Sclerosis and Subacute Sclerosing Panencephalitis. Arch. Neurol.

[29] Górny M.K., Wróblewska Z., Pleasure D., Miller S.L., Wajgt A., Koprowski H. (2009). CSF Antibodies to Myelin Basic Protein and Oligodendrocytes in Multiple Sclerosis and Other Neurological Diseases. Acta Neurol. Scand..

[30] Doolittle D.P., Schweikart K.M. (1977). Myelin Deficient, a New Neurological Mutant in the Mouse. J. Hered..

[31] Popko B., Puckett C., Hood L. (1988). A Novel Mutation in Myelin-Deficient Mice Results in Unstable Myelin Basic Protein Gene Transcripts. Neuron.

[32] Omlin F.X. (1982). Immunocytochemical Localization of Basic Protein in Major Dense Line Regions of Central and Peripheral Myelin. J. Cell Biol..

[33] Readhead C., Takasashi N., Shine H.D., Saavedra R., Sidman R., Hood L. (1990). Role of Myelin Basic Protein in the Formation of Central Nervous System Myelin. Ann. N. Y. Acad. Sci..

[34] Harauz G., Ladizhansky V., Boggs J.M. (2009). Structural Polymorphism and Multifunctionality of Myelin Basic Protein. Biochemistry.

[35] Hoos M.D., Ahmed M., Smith S.O., Van Nostrand W.E. (2007). Inhibition of Familial Cerebral Amyloid Angiopathy Mutant Amyloid β-Protein Fibril Assembly by Myelin Basic Protein. J. Biol. Chem..

[36] Hoos M.D., Ahmed M., Smith S.O., Van Nostrand W.E. (2009). Myelin Basic Protein Binds to and Inhibits the Fibrillar Assembly of Aβ42 in Vitro. Biochemistry.

[37] Liao M.-C., Hoos M.D., Aucoin D., Ahmed M., Davis J., Smith S.O., Van Nostrand W.E. (2010). N-Terminal Domain of Myelin Basic Protein Inhibits Amyloid β-Protein Fibril Assembly. J. Biol. Chem..

[38] Jumper J., Evans R., Pritzel A., Green T., Figurnov M., Ronneberger O., Tunyasuvunakool K., Bates R., Žídek A., Potapenko A. (2021). Highly Accurate Protein Structure Prediction with AlphaFold. Nature.

[39] Martins F., Marafona A.M., Pereira C.D., Müller T., Loosse C., Kolbe K., da Cruz e Silva O.A.B., Rebelo S. (2018). Identification and Characterization of the BRI2 Interactome in the Brain. Sci. Rep..

[40] Pool M.R. (2022). Targeting of Proteins for Translocation at the Endoplasmic Reticulum. Int. J. Mol. Sci..

[41] Shao S., Rodrigo-Brenni M.C., Kivlen M.H., Hegde R.S. (2017). Mechanistic Basis for a Molecular Triage Reaction. Science.

[42] Müller C., Bauer N.M., Schäfer I., White R. (2013). Making Myelin Basic Protein -from mRNA Transport to Localized Translation. Front. Cell. Neurosci..

[43] Shao S., Hegde R.S. (2011). A Calmodulin-Dependent Translocation Pathway for Small Secretory Proteins. Cell.

[44] Smirnova E.V., Rakitina T.V., Ziganshin R.H., Arapidi G.P., Saratov G.A., Kudriaeva A.A., Belogurov A.A. (2021). Comprehensive Atlas of the Myelin Basic Protein Interaction Landscape. Biomolecules.

[45] Senior A.W., Evans R., Jumper J., Kirkpatrick J., Sifre L., Green T., Qin C., Žídek A., Nelson A.W.R., Bridgland A. (2019). Protein Structure Prediction Using Multiple Deep Neural Networks in the 13th Critical Assessment of Protein Structure Prediction (CASP13). Proteins.

[46] de Vries S.J., van Dijk A.D.J., Krzeminski M., van Dijk M., Thureau A., Hsu V., Wassenaar T., Bonvin A.M.J.J. (2007). HADDOCK versus HADDOCK: New Features and Performance of HADDOCK2.0 on the CAPRI Targets. Proteins.

[47] Bates I.R., Libich D.S., Wood D.D., Moscarello M.A., Harauz G. (2002). An Arg/Lys→Gln Mutant of Recombinant Murine Myelin Basic Protein as a Mimic of the Deiminated Form Implicated in Multiple Sclerosis. Protein Expr. Purif..

[48] Fernández-Recio J., Totrov M., Abagyan R. (2004). Identification of Protein–Protein Interaction Sites from Docking Energy Landscapes. J. Mol. Biol..

[49] Barbarese E., Pfeiffer S.E. (1981). Developmental Regulation of Myelin Basic Protein in Dispersed Cultures. Proc. Natl. Acad. Sci. USA.

[50] Happel P., Möller K., Schwering N.K., Dietzel I.D. (2013). Migrating Oligodendrocyte Progenitor Cells Swell Prior to Soma Dislocation. Sci. Rep..

[51] Kudriaeva A., Kuzina E.S., Zubenko O., Smirnov I.V., Belogurov A. (2019). Charge-Mediated Proteasome Targeting. FASEB J..

[52] Dominguez C., Boelens R., Bonvin A.M.J.J. (2003). HADDOCK: A Protein−Protein Docking Approach Based on Biochemical or Biophysical Information. J. Am. Chem. Soc..

[53] Mitternacht S. (2016). FreeSASA: An Open Source C Library for Solvent Accessible Surface Area Calculations. F1000Research.

[54] Jorgensen W.L., Chandrasekhar J., Madura J.D., Impey R.W., Klein M.L. (1983). Comparison of Simple Potential Functions for Simulating Liquid Water. J. Chem. Phys..

[55] Van Der Spoel D., Lindahl E., Hess B., Groenhof G., Mark A.E., Berendsen H.J.C. (2005). GROMACS: Fast, Flexible, and Free. J. Comput. Chem..

[56] Lindorff-Larsen K., Piana S., Palmo K., Maragakis P., Klepeis J.L., Dror R.O., Shaw D.E. (2010). Improved Side-Chain Torsion Potentials for the Amber ff99SB Protein Force Field: Improved Protein Side-Chain Potentials. Proteins.

[57] Horn H.W., Swope W.C., Pitera J.W., Madura J.D., Dick T.J., Hura G.L., Head-Gordon T. (2004). Development of an Improved Four-Site Water Model for Biomolecular Simulations: TIP4P-Ew. J. Chem. Phys..

[58] Berendsen H.J.C., Postma J.P.M., Van Gunsteren W.F., DiNola A., Haak J.R. (1984). Molecular Dynamics with Coupling to an External Bath. J. Chem. Phys..

[59] Parrinello M., Rahman A. (1981). Polymorphic Transitions in Single Crystals: A New Molecular Dynamics Method. J. Appl. Phys..

[60] Van Gunsteren W.F., Berendsen H.J.C. (1988). A Leap-Frog Algorithm for Stochastic Dynamics. Mol. Simul..

[61] Chen G., Andrade-Talavera Y., Tambaro S., Leppert A., Nilsson H.E., Zhong X., Landreh M., Nilsson P., Hebert H., Biverstål H. (2020). Augmentation of Bri2 Molecular Chaperone Activity against Amyloid-β Reduces Neurotoxicity in Mouse Hippocampus in Vitro. Commun. Biol..

