# Supplementary figures and images for: Myelin Basic Protein Attenuates Furin-Mediated Bri2 Cleavage and Postpones Its Membrane Trafficking
Source: Int J Mol Sci. 2024 Feb 23;25(5):2608. doi: 10.3390/ijms25052608 (PMC10932164; doi:10.3390/ijms25052608)

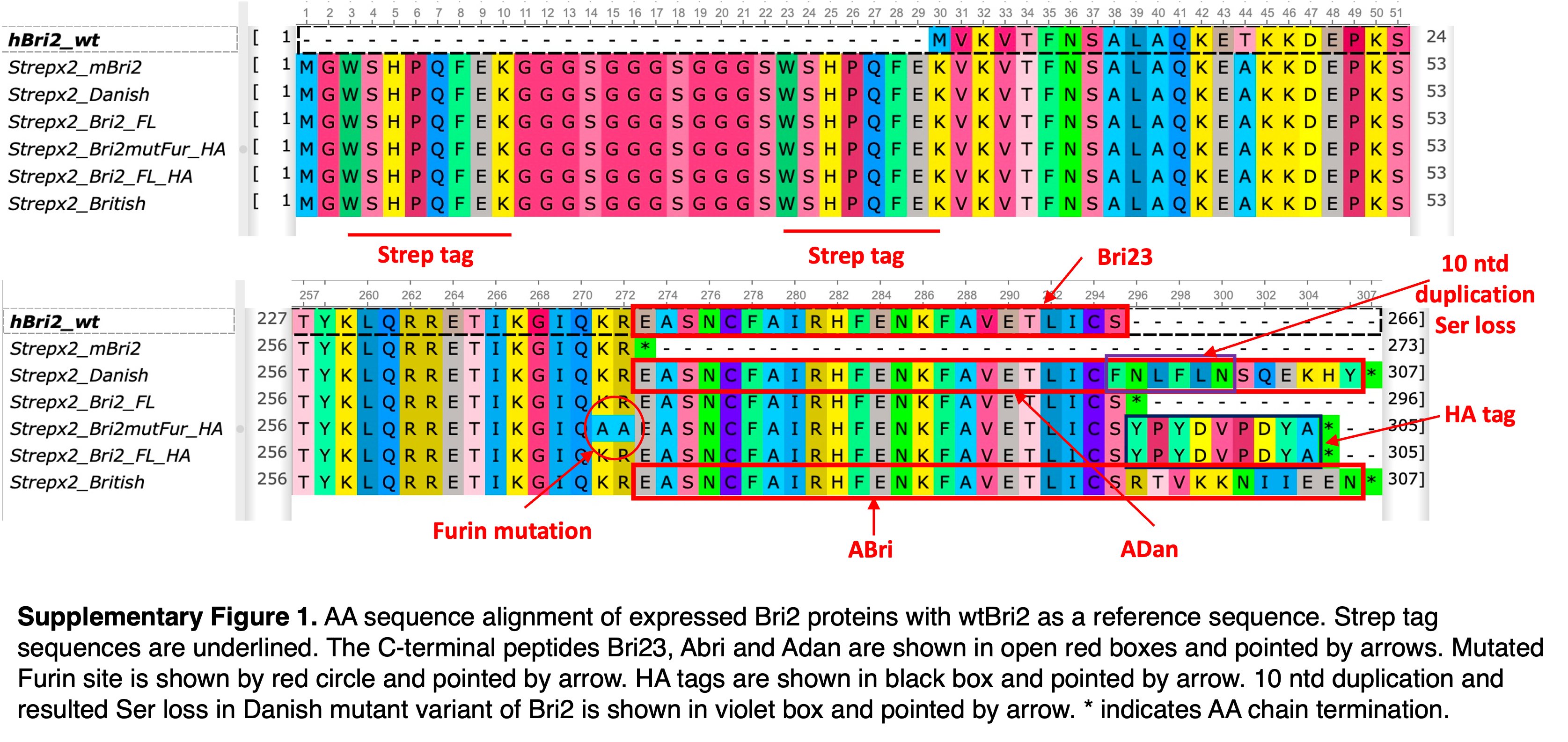

Supplement: Supplementary file 1 [file ijms-25-02608-s001.zip › SupplFigS1.png]
